# Lipid Involvement in Neurodegenerative Diseases of the Motor System: Insights from Lysosomal Storage Diseases

**DOI:** 10.3389/fnmol.2017.00356

**Published:** 2017-11-03

**Authors:** James C. Dodge

**Affiliations:** Neuroscience Therapeutic Area, Sanofi, Framingham, MA, United States

**Keywords:** galactolipids, gangliosides, glycogen, glycosphingolipids, lysosome, neuromuscular disease, motor neuron disease, sphingolipid

## Abstract

Lysosomal storage diseases (LSDs) are a heterogeneous group of rare inherited metabolic diseases that are frequently triggered by the accumulation of lipids inside organelles of the endosomal-autophagic-lysosomal system (EALS). There is now a growing realization that disrupted lysosomal homeostasis (i.e., lysosomal cacostasis) also contributes to more common neurodegenerative disorders such as Parkinson disease (PD). Lipid deposition within the EALS may also participate in the pathogenesis of some additional neurodegenerative diseases of the motor system. Here, I will highlight the lipid abnormalities and clinical manifestations that are common to LSDs and several diseases of the motor system, including amyotrophic lateral sclerosis (ALS), atypical forms of spinal muscular atrophy, Charcot–Marie–Tooth disease (CMT), hereditary spastic paraplegia (HSP), multiple system atrophy (MSA), PD and spinocerebellar ataxia (SCA). Elucidating the underlying basis of intracellular lipid mislocalization as well as its consequences in each of these disorders will likely provide innovative targets for therapeutic research.

## Overview of Lysosomal Storage Diseases

**Figure 1:**
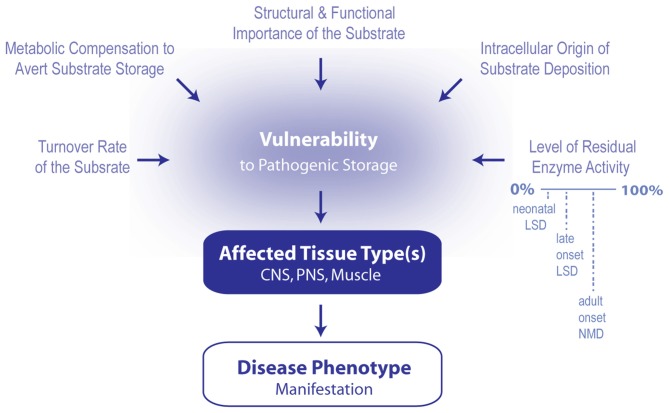
Model summarizing the contributing factors that determine which phenotype arises following the induction of lipid cacostasis. A number of factors likely determine which cell types are particularly vulnerable to the pathogenic storage of a given lipid. These include cellular variations in lipid turnover (rate of lipid synthesis, metabolism and transport), metabolic compensatory responses (e.g., shuttling of metabolic precursors of the accumulating lipid to alternative metabolic pathways) to avert storage, and cellular differences in the relative role that the lipid plays in the cell’s structure and signaling pathways. Pathogenic storage of the lipid is also likely affected by where lipid deposition originates within the cell (i.e., in which organelle) and the level of residual enzyme activity of the affected protein. The latter two variables also likely influence which type of disease (e.g., early onset lysosomal storage disease (LSD), late onset LSD or adult onset neuromuscular disease (NMD) manifests. Primarily affected cell types determined which tissue types (i.e., central nervous system (CNS), peripheral nervous system (PNS) and muscle) are affected first; and therefore, which disease symptoms first develop.

Lysosomal storage diseases (LSDs) are a subgroup of genetic metabolic diseases that feature disrupted lysosomal homeostasis (i.e., lysosomal cacostasis) often initiated by a deficiency in a soluble acidic hydrolase that resides within the lumen of the lysosome. In some cases, defects in lysosomal membrane proteins or in non-enzymatic lysosomal proteins are the underlying cause of disease. In all LSDs there is a progressive and relentless accumulation of undegraded macromolecules and monomeric compounds inside the organelles of the endosomal-autophagic-lysosomal system (EALS). Typically, the compound that accumulates during the course of the disease is used to classify the disorder. However, in most LSDs, more than one compound accumulates, and in some disorders, the stored material is often heterogeneous (Neufeld, 1991; Ballabio and Gieselmann, 2009; Platt et al., 2012). Although individual LSDs are relatively rare, these disorders have a combined incidence of 1 per 5000 live births (Meikle et al., 1999; Fuller et al., 2006). Of the approximate 50 LSDs that have been identified thus far, more than 70% feature significant central nervous system (CNS) involvement (Wraith, 2004), which is not surprising given that lysosomes recycle cellular debris and they are vital to maintaining cellular homeostasis (Sardiello et al., 2009; Palmieri et al., 2011). The neuropathological features of LSDs include neurodegeneration, neuroinflammation, astrogliosis, cerebrovascular abnormalities and demyelination. At the cellular level, lysosomal cacostasis is often accompanied by reduced autophagic flux, impaired endosomal clearance, disrupted calcium homeostasis in the endoplasmic reticulum (ER) and mitochondria, increased reactive oxygen species generation and the progressive accumulation of ubiquitinated proteins in the cytoplasm (Ballabio and Gieselmann, 2009; Platt et al., 2012; Pastores and Maegawa, 2013). For each disease, neurological symptoms are often determined by the nature of the storage material, which dictates the initial locus of metabolic derangement and the rate at which storage pathology cascades throughout the rest of the CNS (Walkley, 2009). The specific disease pattern likely occurs for a number of reasons (Figure 1). Different neuronal and glial subtypes may synthesize the stored compound at different rates, may be dependent to variable extents on the stored material for normal function, and may have disparate compensatory responses to avert pathogenic storage. For example, in Niemann Pick Type A disease, ataxia manifests when the stored material (i.e., sphingomyelin) rapidly accumulates within the cerebellum and triggers Purkinje cell death (Sarna et al., 2001). In some LSDs, clinical symptoms are evident shortly after birth, whereas in others, a disease phenotype does not appear until early childhood or later in life. The age of symptom onset and the degree of clinical manifestation may vary widely, even between family members carrying identical mutations. Typically, disease severity is correlated with the level of residual enzyme activity, with lower levels resulting in more aggressive and pernicious symptoms. Disease progression in neuropathic LSDs is often rapidly fatal, especially in patients who present with the disease during the neonatal period. Thus, the observed disease phenotype is also a function of the individual’s level of neurodevelopment achieved prior to substrate storage becoming pathogenic. Unsurprisingly, mild or late onset lysosomal cacostasis is increasingly recognized as an underlying cause of neurodegeneration in diseases that are ostensibly unrelated to LSDs. Here, I will discuss the potential pathogenic role played by lysosomal hydrolases and their respective lipid substrates in several diseases of the motor system.

## Gaucher Disease, Glucocerebrosidase and Glucosylceramide

**Figure 2:**
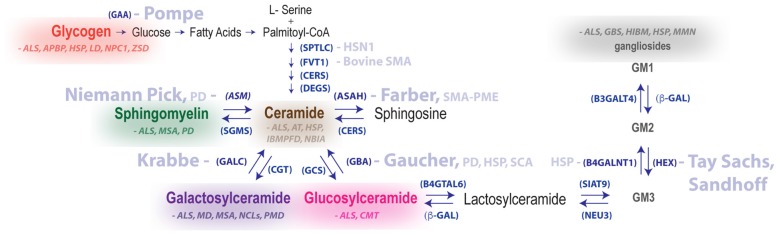
Summary of metabolic pathways associated with LSDs and neurodegenerative diseases of the motor system. Disease names in light blue are genetically linked to defective hydrolases. Glycogen and lipid substrate (sphingomyelin, ceramide, galactosylceramide, glucosylceramide and gangliosides) levels are altered in the nervous system for a number of diseases featuring motor dysfunction (substrates and diseases shown in the same color are biochemically linked). Enzyme names are shown in parentheses. Abbreviations: GAA, acid alpha-glucosidase; ASM, acid sphingomyelinase; APBD, adult polyglucosan body disease; ALS, amyotrophic lateral sclerosis; ASAH1, acid ceramidase; AT, ataxia telangiectasia; B4GALNT1, b-1,4-N-acetyl-galactosaminyl transferase 1, GM2 synthase; b-GAL, b-galactosidase; B4GALT6, b-1,4-galactosyltransferase 6, lactosylceramide synthase, CGT, ceramide galatosyltransferase; CERS, ceramide synthase; CMT, Charcot–Marie–Tooth disease; DEGS, dihydroceramide desaturase; B3GALT4, ganglioside galactosyltransferase; GALC, galactocerebrosidase; GCS, glucosylceramide synthase, GBA, glucocerebrosidase; GBS, Guillain-Barré syndrome; HEX, hexosaminidase; HIBM, hereditary inclusion body myopathy; HSN1, hereditary sensory neuropathy type 1; HSP, hereditary spastic paraplegia; FVT1, 3-ketodihydrosphingosine reductase, LD, Lafora disease; HMSN, motor and sensory neuropathy, MMN, multifocal motor neuropathy; MSA, multiple system atrophy; NEU3, neuraminidase 3; NBIA, neurodegeneration with brain iron accumulation; NCLs, neuronal ceroid lipofuscinoses; NPC1, Niemann-Pick Type C disease; IBMPFD, Paget’s disease of bone, frontotemporal dementia; PD, Parkinson disease; PMD, Pelizaeus–Merzbacher disease; SPTLC1, serine palmitoyltransferase long chain base subunit-1; SIAT9, sialyltransferase 9, GM3 synthase SGMS, sphingomyelin synthase; SMA-PME, spinal muscular atrophy myoclonic epilepsy; ZSD, Zellweger spectrum disorder.

Glucosylceramide (GlcCer) is essential for normal brain development and function. Disruption of GlcCer synthesis in the CNS of mice leads to dendritic shortening, structural abnormalities in axons and myelin, motor dysfunction and a reduced lifespan (Jennemann et al., 2005). Autosomal recessive mutations in glucocerebrosidase (GBA), a lysosomal enzyme that hydrolyzes GlcCer to form glucose and ceramide, leads to Gaucher disease (GD), which has three clinical forms distinguished by the presence and degree of neurological complications (Figure 2). Type I GD patients typically present with systemic GlcCer accumulation, whereas GD patients with more severe deficiencies in GBA (i.e., types II and III) feature early neurological manifestations (Cox and Schofield, 1997). In type II disease, which is typically fatal before 2 years of age, neurological symptoms such as oculomotor apraxia, strabismus, hypertonia and retroflexion of the head become evident within the first few months of life. Manifestations of type III disease include horizontal supranuclear gaze palsy, myoclonic epilepsy, ataxia, spasticity and dementia, and patients with this type usually succumb to the disease in early adulthood (Nagral, 2014). Notably, natural history studies recently captured the development of a late-onset neuropathic phenotype in patients with GD. Specifically, these studies detected neurological complications resembling Parkinson disease (PD) in aged Gaucher patients with a mild form of the disease (Neudorfer et al., 1996) and were some of the first to suggest that lysosomal dysfunction may also play a role in some of the more common neurodegenerative diseases. Today, heterozygous mutations in GBA are now recognized as a leading PD risk factor (Sidransky et al., 2009; Nalls et al., 2013) and are also associated with accelerated cognitive decline in PD (Liu et al., 2016). Moreover, PD patients without mutations in GBA can also exhibit lower enzyme activity in the CNS (Gegg et al., 2012), thus further implicating the lysosomal enzyme in the pathogenesis of PD.

A deficiency in GBA enzyme activity also appears to play a role in spinocerebellar ataxia (SCA) and hereditary spastic paraplegia (HSP). SCAs comprise a large group of heterogeneous inherited neurodegenerative disorders that feature progressive cerebellar ataxia with oculomotor dysfunction, dysarthria, pyramidal signs, extrapyramidal signs, pigmentary retinopathy, peripheral neuropathy and cognitive impairment. SCAs are currently classified into 40 different (SCA1–40) subtypes (Harding, 1993; Schöls et al., 1997). HSPs are a group of rare inherited heterogeneous neurological diseases that are divided into pure forms, which manifest as pyramidal signs leading to lower-limb spasticity, and complex forms, which may also feature spastic ataxia, dysarthria, dystonia, cognitive impairment, optic tract atrophy and hearing loss. To date, 76 loci and 59 corresponding spastic paraplegia genes (SPGs) are associated with HSPs. Many of the pathogenic mutations of SPGs lead to the progressive degeneration of upper motor neurons and the associated corticospinal tracts. However, in some cases, lower-limb muscle atrophy and fasciculations are observed, thus suggesting lower motor neuron involvement (Harding, 1981; Klebe et al., 2015). In contrast to PD, which is linked to a deficiency in lysosomal GBA activity, loss-of-function mutations in GBA2, a microsomal and non-lysosomal GBA, are associated with an autosomal dominant form of SCA (Votsi et al., 2014; Sultana et al., 2015; Synofzik and Schüle, 2017) and an autosomal recessive form of HSP featuring cognitive impairment (Martin et al., 2013; Sultana et al., 2015; Yang et al., 2016). Consistent with these genetic observations, the knockdown of the GBA2 orthologous gene in zebrafish results in abnormal motor behavior and the shortening of motor neuron axons (Martin et al., 2013).

GlcCer, the primary substrate of GBA, also appears to participate in the pathogenesis of amyotrophic lateral sclerosis (ALS) and CMT type III (CMT3). ALS is a progressive, paralytic disorder characterized by degeneration of motor neurons in the brain and spinal cord. To date, 25 genes are associated with the development of ALS (Ghasemi and Brown, 2017). ALS initially manifests as focal weakness but then spreads inexorably until most muscles are involved, including the diaphragm. Respiratory paralysis, which typically occurs 3–5 years after symptom onset is the main cause of death. The clinical presentation of ALS is heterogeneous. There are bulbar and spinal-onset forms of ALS (Brown and Al-Chalabi, 2017) and 15%–20% of ALS patients feature behavioral abnormalities and cognitive impairment that often leads to dementia (Bang et al., 2015). Several lines of evidence suggest that GlcCer modulates the disease course in ALS. For example, GlcCer levels are significantly elevated in the spinal cords of sporadic ALS (sALS) patients and in SOD1^G93A^ mice, a familial model of ALS. Moreover, changes in GlcCer levels are associated with concomitant fluctuations in acidic (GBA) and neutral (GBA2) enzyme activities within the spinal cord (Dodge et al., 2015). Intriguingly, inhibition of GlcCer synthesis in the CNS, an approach that is efficacious in mouse models of neuropathic GD (Cabrera-Salazar et al., 2012) and Gaucher-related PD (Sardi et al., 2017), sped up disease progression in SOD1^G93A^ mice (Dodge et al., 2015; Henriques et al., 2015). This effect is likely mediated at the level of the CNS because treatment with a non-CNS penetrant inhibitor of GlcCer synthesis had no effect on disease course (Dodge et al., 2015). Collectively, these findings suggest that the reductions in GBA activity that occur in SOD1^G93A^ mice during the disease are a part of a compensatory response to slow progression (Dodge et al., 2015). Indeed, enhancing low-level inhibition of GBA activity in SOD1^G93A^ mice preserved the integrity of their motor units (Henriques et al., 2017). CMT is the most common hereditary neuromuscular disorder, with an estimated prevalence of 40 cases per 100,000 individuals (Martyn and Hughes, 1997). Although CMT is genetically heterogeneous, with 25 genes linked to the disorder thus far, it features a common clinical phenotype that arises from the involvement of motor and sensory nerves of the peripheral nervous system (PNS). CMT is also known as motor and sensory neuropathy (HMSN). Disease features and symptoms of CMT include structural foot deformities, muscle wasting in the lower legs and feet, frequent tripping, a loss of manual dexterity, cold temperature intolerance in the extremities, fatigue, sleep apnea and hearing loss (Pareyson and Marchesi, 2009). In contrast to neuromuscular diseases (NMDs) featuring elevated GlcCer levels in the CNS, CMT3 disease symptoms are associated with reduced GlcCer levels in the PNS (Yao and Dyck, 1981). Collectively, these diseases suggest that the intracellular titration of GlcCer is important for multiple components of the neuromuscular axis.

## GM2 Gangliosidosis, β-Hexosaminidase and Gangliosides

The successive addition of galactose and sialic acid moieties to GlcCer results in the synthesis of anionic gangliosides (e.g., GM3, GM2 and GM1; Yu et al., 2004). Gangliosides are especially abundant in the CNS and are involved in metabolism, growth factor signaling, oligodendrocyte differentiation, neuroinflammation, angiogenesis and apoptosis (Yim et al., 1994; Yu et al., 2004; Chatterjee and Pandey, 2008; Inokuchi, 2009; Prokazova et al., 2009; Nordström et al., 2013). GM2 gangliosidosis is a rare and heterogeneous autosomal recessive LSD caused by a deficiency in β-hexosaminidase A, a combined deficiency in β-hexosaminidases A and B, or a deficiency in the non-catalytic GM2 activator (Maegawa et al., 2006). The enzyme β-hexosaminidase comprises two major isoenzymes, β-hexosaminidase A and β-hexosaminidase B. β-Hexosaminidase A is a heterodimeric isoenzyme made up of two different subunits, α and β, that are encoded by the genes *HEXA* and *HEXB*. It catalyzes the removal of β-*N*-acetylgalactosamine from the ganglioside GM2 to generate GM3. β-Hexosaminidase B is composed of two identical β subunits (β2) and does not stimulate the degradation of GM2. Deficiency of the GM2 activator protein, which mediates the interaction between the water-soluble β-hexosaminidase A and its membrane-embedded substrate, GM2 ganglioside, causes the AB variant of GM2 gangliosidosis (Maegawa et al., 2006).

Mutations of the *HEXA* gene encoding the α subunit cause deficiency of β-hexosaminidase A and result in Tay-Sachs disease, the most common form of which manifests as muscular weakness by 3–6 months of age (Figure 2). As the disease progresses, children with Tay-Sachs experience seizures, visual impairment, hearing loss and paralysis. The disease is usually fatal by 3 years of age. Patients with milder disease variants do not develop symptoms until childhood, adolescence or even adulthood. Patients with late-onset Tay-Sachs disease exhibit cerebellar ataxia, dysarthria, anterior horn degeneration and a clinical phenotype that often mimics a spectrum of NMDs including atypical Friedreich ataxia, juvenile-onset spinal muscular atrophy and motor neuron disease (MND; Johnson, 1981; Johnson et al., 1981; Willner et al., 1981; Argov and Navon, 1984; Mitsumoto et al., 1985; Parnes et al., 1985). Mutations of the *HEXB* gene, encoding the β-subunit, cause deficiency of both isoenzymes (β-hexosaminidase A and β-hexosaminidase B), leading to Sandhoff disease, which is, in clinical aspects, virtually indistinguishable from Tay-Sachs disease. Similar to patients with late-onset Tay-Sachs disease, individuals with adult-onset Sandhoff disease present with a clinical phenotype that often masquerades as MND (Hancock et al., 1985; Rubin et al., 1988; Banerjee et al., 1994).

Several lines of evidence suggest that fluctuations in β-hexosaminidase enzyme activity and gangliosides also contribute to the pathogenesis of a number of movement disorders including ALS, HSP, multifocal motor neuropathy (MMN), PD, Guillain-Barré syndrome (GBS) and hereditary inclusion body myopathy (HIBM). For example, several labs have reported that Hex mRNA is up regulated in the spinal cords of SOD1^G93A^ mice (Olsen et al., 2001; Lobsiger et al., 2007; Baker et al., 2015; Dodge et al., 2015). Moreover, acidic (lysosomal) and neutral (cytoplasmic) β-hexosaminidase enzyme activities are elevated not only in the spinal cords of SOD1^G93A^ mice but also in the spinal cord gray and white matter of sALS patients (Dodge et al., 2015). A subsequent study confirmed that lysosomal β-hexosaminidase enzyme activity is elevated in the spinal cords of SOD1^G93A^ mice (Baker et al., 2015). The potential involvement of gangliosides in ALS was initially highlighted in early clinical studies that reported serum auto-antibodies to GM1 and GM2, along with the detection of unusual gangliosides in the brain and spinal cords of ALS patients (Dawson and Stefansson, 1984; Rapport et al., 1985; Salazar-Grueso et al., 1990). Consistent with increased β-hexosaminidase activity, GM3, the ganglioside that is generated from the hydrolysis of GM2 by β-hexosaminidase, is elevated in the spinal cords of SOD1^G93A^ mice and sALS patients (Dodge et al., 2015). Further increasing β-hexosaminidase activity in SOD1^G93A^ mice (to increase GM3 levels) had no impact on the disease course; however, intracerebroventricular infusion of exogenous GM3 modestly slowed disease progression (Dodge et al., 2015). Disrupted ganglioside homeostasis is also implicated in HSP and MMN. Mutations in beta-1,4-N-acetyl-galactosaminyl transferase 1 (B4GALNT1; GM2 synthase) result in early-onset HSP, intellectual disability, cerebellar ataxia, peripheral neuropathy and cortical atrophy (Boukhris et al., 2013; Harlalka et al., 2013). MMN is an ALS mimic that manifests as slowly progressive asymmetrical weakness of the distal limbs associated with high serum levels of IgM antibodies against the ganglioside GM1 (Vlam et al., 2011). GM1 also appears to play a role in the pathogenesis of PD. For example, PD patients also feature high titer serum IgM anti-GM1 antibodies (Zappia et al., 2002) and in the substantia nigra of some PD patients GM1 staining is diminished near α-synuclein aggregates and neurons with reduced levels of tyrosine hydroxylase (TH; Wu et al., 2012). Mice that are genetically deficient in GM1 display several features consistent with PD including the loss of TH-positive cells, lower striatal dopamine levels, an accumulation of α-synuclein aggregates and impaired motor function (Wu et al., 2011). Moreover, treatment with exogenous GM1 is beneficial in multiple preclinical models of PD (Schneider, 1998) and a randomized placebo control trial reported that systemic administration of GM1 improved functional outcome in PD patients (Schneider et al., 1998).

Gangliosides are also important to peripheral nerve and normal muscle function. For example, GBS, the most frequent cause of acute neuromuscular paralysis, is associated with pathogenic changes in ganglioside homeostasis. Antibodies to ganglioside GM1 are present in 14%–50% of patients with GBS and are more common in cases with severe axonal degeneration associated with any subtype (Gregson et al., 1993; Hughes et al., 1999). Notably, anti-GM1 IgG from patients with GBS blocks muscle action potentials in a muscle-spinal cord co-culture (Yuki et al., 2004) and attaches to the nodes of Ranvier in peripheral nerves to activate the complement cascade (Paparounas et al., 1999). GM3 is the most abundant ganglioside in muscle, representing almost 70% of total ganglioside levels in muscle membranes (Müthing and Cacić, 1997), and it regulates muscle cell differentiation and proliferation (Cambron and Leskawa, 1994). Not surprisingly, HIBM, an autosomal recessive muscle disorder characterized by adult-onset muscle weakness in upper and lower limbs, is associated with mutations in the UDP-N-acetylglucosamine 2-epimerase/N-acetylmannosamine kinase gene (Eisenberg et al., 2001), which regulates sialic acid synthesis (Hinderlich et al., 1997), and thus, the synthesis of GM3 (Wang et al., 2006). Indeed, the muscles of a mouse model of HIBM express significantly lower GM3 levels (Paccalet et al., 2010). Collectively, these studies indicate that β-hexosaminidase and gangliosides play critical functional roles at multiple levels of the neuromuscular axis and contribute to a number of NMD phenotypes when disrupted.

## Farber Disease, Ceramidase and Ceramide

Ceramide is the hydrophobic backbone of GlcCer, gangliosides and sphingolipids. In mammals, ceramide comprises a family of more than 200 structurally distinct molecules, and its metabolism is controlled by 28 different enzymes (Hannun and Obeid, 2011). Ceramide plays a bioactive role in a number of signal transduction pathways. For example, various forms of cellular stress lead to intracellular ceramide accumulation and the subsequent induction of apoptotic cell death (Kolesnick and Krönke, 1998). Farber lipogranulomatosis, or Farber disease (FD), is an autosomal recessive LSD caused by a deficiency in acid ceramidase (ASAH1), the lysosomal enzyme that catalyzes the hydrolysis of ceramide (Figure 2). FD is divided into seven types with varying degrees of neurological involvement (Ehlert et al., 2007). Patients with type I disease, the classic form, feature early subcutaneous nodules, joint stiffness, hoarseness due to laryngeal involvement and progressive neurological and pulmonary deterioration. In contrast, type II and III patients display limited CNS symptoms but still fail to thrive because of joint pain and respiratory involvement. In type IV patients, severe neurological deterioration is observed during the neonatal period, whereas in type V patients, progressive CNS dysfunction manifests sometime between 1 and 2.5 years of age (Pavone et al., 1980; Antonarakis et al., 1984; Eviatar et al., 1986). CNS symptoms of FD include tetraplegia, loss of speech, myoclonia, seizures and mental retardation. Patients with types VI and VII disease also feature CNS involvement; however, in these variants, additional lysosomal enzymes (e.g., GBA, β-hexosaminidase and galactocerebrosidase (GALC)) are also impaired (Fusch et al., 1989; Schnabel et al., 1992). In most cases, the deterioration of the CNS limits survival to the first few years of life.

Hematopoietic stem cell transplantation (HSCT) alleviates a number of symptoms of peripheral FD disease but does not prevent progressive neurological deterioration. Interestingly, in some cases, FD patients progress to an MND phenotype following HSCT (Cappellari et al., 2016), suggesting that if the systemic component of the disease is addressed while leaving the CNS untreated, a new phenotype arises. Moreover, mutations in ASAH1 are also associated with a form of spinal muscular atrophy that also features progressive myoclonic epilepsy (SMA-PME). Unlike SMA itself, the MND phenotype in SMA-PME patients is not due to a mutation in the survival of motor neuron 1 (SMN1) gene (Jankovic and Rivera, 1978; Zhou et al., 2012; Gan et al., 2015; Rubboli et al., 2015). In most patients, muscle weakness manifests within 1–10 years, followed by the development of seizures. Disease severity and progression varies significantly between patients, and some patients with SMA-PME also develop hearing loss, action tremor, cognitive impairment and cerebral atrophy. Mutations in ASAH1 found in SMA-PME patients result in residual enzyme activity between 5% and 30% of control levels (Zhou et al., 2012; Dyment et al., 2014). Consistent with these findings, a NMD phenotype is also observed in animal models with impaired ASAH1 activity. For example, knockdown of the ASAH1 ortholog in zebrafish with a morpholino antisense oligonucleotide leads to increased apoptosis and a marked loss of motor neuron axonal branching in the spinal cord (Zhou et al., 2012). Although complete knockout of ASAH1 in mice is embryonic lethal (Li et al., 2002), knock-in of a severe ASAH1 gene mutation into the corresponding mouse sequence is associated with delayed growth, progressive weight loss, lethargy, and general dystrophy, as well as a progressive loss in grip strength that ends in death between 7 and 13 weeks of age (Alayoubi et al., 2013).

In addition to SMA-PME, several lines of evidence suggest that ceramide cacostasis plays a role in a number of other NMDs. For instance, elevated ceramide levels are found in the spinal cords of sALS patients and SOD1^G93A^ mice (Cutler et al., 2002; Dodge et al., 2015). Notably, promoting ceramide accumulation by inhibiting glucosylceramide synthase (GCS) exacerbates the disease in SOD1^G93A^ mice (Dodge et al., 2015). An aberrant increase in ceramide is also implicated in the pathogenesis of ataxia telangiectasia (AT) and hereditary sensory neuropathy type 1 (HSN1). AT is an autosomal recessive disease characterized by cerebellar ataxia, telangiectasias, apraxia and progressive neurological deterioration. AT is caused by homozygous or compound heterozygous mutations in the ATM gene, which inhibits DNA damage-induced apoptosis through repression of ceramide synthase (Jaspers et al., 1988). Cell lines expressing mutant ATM feature enhanced ceramide synthase activity and apoptosis (Liao et al., 1999). HSN1 is the most common inherited disorder of peripheral sensory neurons that features progressive degeneration of dorsal root ganglia and motor neurons, usually sometime during the 2nd or 3rd decade of life. Symptoms of HSN1 include sensory loss followed by distal muscle wasting and weakness. HSN1 is associated with mutations in the gene encoding serine palmitoyltransferase long chain base subunit-1 (SPTLC1), the rate-limiting enzyme in de novo ceramide synthesis. Interestingly, expression of mutant SPTLC1 from affected individuals in lymphoblast cell lines significantly *increased* GlcCer synthesis, thus suggesting a toxic up regulation of ceramide synthesis (Dawkins et al., 2001).

Disrupted ceramide synthesis is also featured in a number of NMDs, thus suggesting that ceramide levels must be closely titrated to ensure normal motor function. For example, autosomal recessive mutations in fatty acid 2-hydroxylase (FA2H), which catalyzes the hydroxylation of ceramide at position 2 of the N-acyl chain, result in HSP type 35, which is characterized by early-onset progressive spasticity, ataxia, dystonia and cognitive decline (Edvardson et al., 2008). Defective FA2H also leads to object neurodegeneration with brain iron accumulation (NBIA), a spectrum disorder with phenotypes ranging from infantile with early death to adult-onset parkinsonism-dystonia. Patients with NBIA exhibit cerebellar atrophy, spastic quadriparesis, ataxia, dystonia and episodic neurological decline (Kruer et al., 2010). A deficiency in ceramide synthesis is also observed in two mouse strains that feature cerebellar ataxia and Purkinje cell death. Flincher and toppler mice have spontaneous autosomal recessive mutations in ceramide synthase 1, an enzyme that regulates the de novo synthesis of certain ceramide species (Zhao et al., 2011). Moreover, a mutation in 3-ketodihydrosphingosine reductase (FVT1), which catalyzes the 2nd step in de novo ceramide synthesis, is associated with bovine spinal muscular atrophy (Krebs et al., 2007). Ceramide accumulation in the muscle may also contribute to phenotypes found in NMD. Mutations in the valosin-containing protein (VCP) gene are associated with a heterogeneous group of disorders including HIBM, Paget’s disease of bone, frontotemporal dementia (IBMPFD) and ALS. Patients with IBMPFD exhibit progressive muscular atrophy and weakness and die from cardiac and respiratory failure. Notably, VCP mutant mice display a phenotype that is similar to the IBMPFD phenotype associated with ceramide accumulation in the muscle (Llewellyn et al., 2014). Collectively, these findings indicate that maintaining ceramide in an optimal homeostatic range (or eustasis) is important for multiple functional aspects of the neuromuscular axis.

## Krabbe Disease, Galactocerebrosidase and Galactosylceramide

Galactosylceramide (GalCer) is synthesized when galactose is added to the 1-hydroxyl moiety of ceramide. GalCer and its sulfated form, sulfatide, account for approximately one-third of the lipid content in myelin (Norton et al., 1975). The high content of galactolipids is believed to provide myelin with its structural stability (i.e., compacted sheath) and curvature. During development, GalCer also modulates the differentiation and maturation of oligodendrocytes, the initiation of myelination and the establishment of axo-glial interactions at the nodes of Ranvier (Marcus and Popko, 2002). Krabbe disease (KD) or globoid cell leukodystrophy (GLD) is due to autosomal recessive mutations in GALC, a lysosomal enzyme that catalyzes the hydrolysis of GalCer and galactosylsphingosine (Figure 2). Unlike most LSDs, the primary substrate (in this case GalCer) does not rapidly accumulate because its degradation is also catalyzed by β-galactosidase (β-GAL), an enzyme that also hydrolyzes lactosylceramide and the ganglioside GM1 (Kobayashi et al., 1985). Indeed, β-GAL deficiency is implicated in some patients with KD (Wenger et al., 1974). Substrate accumulation in KD leads to the formation of globoid cells, which are large multinucleated macrophages surrounding cerebral blood vessels in white matter, and leukodystrophy, a state of demyelination and gliosis (Miyatake and Suzuki, 1972). At the cellular level, the recruitment of signaling proteins to lipid rafts is impaired, endocytosis is disrupted and axonal transport is severely compromised during disease (Nogueira-Rodrigues et al., 2016). There are both infantile and late-onset forms of KD. The infantile form of KD occurs in 85%–90% of all patients, manifests prior to 6 months of age and is usually fatal before the child’s 2nd birthday. Features of infantile KD include irritability, fever, limb stiffness, seizures, difficulty in eating, slowed cognitive and motor development, and growth retardation. The remaining cases of KD have a later onset, commonly differentiated as late infantile (6 months to 3 years), adolescent (3–8 years) or adult-onset (Wenger et al., 2000). Late-onset forms of KD are often clinically heterogeneous and less severe. For example, these patients appear normal until weakness, spastic paraparesis, vision loss, and cognitive decline become evident (Tappino et al., 2010). In some instances, adult-onset KD mimics MND (Henderson et al., 2003) or HSP (Bajaj et al., 2002). Similar to humans, a deficiency in GALC activity also leads to hypomyelination and lower nerve conduction velocities in mice (Potter et al., 2013) and primates (Weimer et al., 2005).

Galactolipid cacostasis resulting from either impaired galactolipid transport or synthesis is also associated with progressive motor dysfunction. For example, some of the protein products of genes associated with neuronal ceroid lipofuscinoses (NCLs), the most common group of inherited childhood neurodegenerative disorders, appear to modulate the intracellular distribution of GalCer. CNS symptoms of NCLs include blindness, motor incoordination, seizures and cognitive impairment. NCLs are associated with mutations in a number of different genes (CLN1–CLN14) that produce protein products that reside within varying cellular locations including the lysosome, ER and the vesicular membranes of the cytosol. In most instances, the function of these proteins is poorly characterized or remains unknown (Cárcel-Trullols et al., 2015). Emerging evidence suggests that some of these CLN genes may also contribute to other neurodegenerative diseases. For example, mutations in CLN1 (palmitoyl-protein thioesterase 1; PPT-1) and CLN2 (tripeptidyl-peptidase 1; TPP-1) are also associated with neuronal loss in the substantia nigra in adults (Nijssen et al., 2003), early onset frontotemporal dementia (Jeung et al., 2015) and juvenile onset forms of ataxia (SCA type 7; SCAR7; Sun et al., 2013). Notably, the mutations in CLN3 that are linked to a juvenile form of NCL result in reduced GalCer and sulfatide binding and impaired transport of GalCer from the ER/Golgi to lipid rafts. Additional experiments indicate that CLN6 and CLN8 also bind GalCer (Rusyn et al., 2008). Interestingly, a naturally occurring mutation in the murine CLN8 gene that results in an MND phenotype (Messer and Flaherty, 1986; Bronson et al., 1993) is associated with an early impairment in GalCer synthesis and oligodendrocyte maturation (Kuronen et al., 2012).

Several lines of evidence suggest that galactolipid cacostasis may also contribute to the pathogenesis of additional diseases affecting the motor system including ALS, multiple system atrophy (MSA), Pelizaeus–Merzbacher disease (PMD) and Menkes disease. For example, in the spinal cords of sALS patients, GalCer levels are elevated, and the enzyme activities of GALC and β-GAL, both in the lysosome and cytoplasm, are altered. The latter finding suggests that galactolipid metabolism is disrupted in multiple cellular organelles in ALS. Similar to sALS patients, aberrant fluctuations in GalCer, GALC and β-GAL also manifest during the disease course in SOD1^G93A^ mice (Dodge et al., 2015). MSA is a rapidly progressive neurodegenerative disease characterized by Parkinsonism, cerebellar ataxia, autonomic failure, and the accumulation of alpha-synuclein in oligodendrocytes. Recent findings suggest that myelin instability in MSA is associated with a significant reduction in GalCer levels (Don et al., 2014). Similarly, both PMD, a leukodystrophy associated with mutations in proteolipid protein 1 (Trofatter et al., 1989), and Menkes disease, a lethal disorder of copper metabolism featuring neurodegeneration, hypotonia, weakness and spasticity, are also associated with significant reductions in myelin galactolipid levels (Lou et al., 1974; Witter et al., 1980). PMD is an unusual leukodystrophy because it results from the improper formation of myelin rather than from demyelination, the predominant cause of most leukodystrophies. Features of PMD include hypotonia, nystagmus, spasticity, ataxia and the development of choreoathetotic movements later in life. Patients with PMD typically deteriorate slowly until death in mid-adulthood (Inoue, 2005).

## Niemann-Pick Disease, Sphingomyelinase and Sphingomyelin

Niemann-Pick disease (NPD) is an eponym referring to a disease in which patients feature varying degrees of lipid storage, hepatosplenomegaly, respiratory impairment and CNS involvement. NPD is caused by two different metabolic abnormalities. Patients with the type A or type B disease have a deficiency in sphingomyelin phosphodiesterase 1 (SMPD1), also known as acid sphingomyelinase (ASM), the lysosomal enzyme that hydrolyzes sphingomyelin to form ceramide and phosphocholine (Figure 2; Kampine et al., 1967). NPD type C patients also have deficiencies in ASM and moderate accumulation of sphingomyelin; however, this is secondary to the defect in cholesterol esterification (Pentchev et al., 1984, 1985) that is due to autosomal recessive mutations in two distinct cholesterol-binding proteins, Niemann-Pick C1 (NPC1) and NPC2 (Carstea et al., 1997; Naureckiene et al., 2000). Additional secondary metabolic abnormalities of NPD type C include reduced GBA enzyme activity and the accumulation of GM2 and GM3 (Pentchev et al., 1980a,b).

Type A and type B NPD form a continuum of phenotypes varying from acute neurological to chronic neurological and chronic non-neurological. CNS abnormalities, including hypotonia, motor incoordination, akinesia, spasticity and a loss of deep tendon reflexes, typically manifest between 5 and 10 months of age in NPD type A patients. Sadly, children typically succumb to the disease prior to their 3rd birthday. Type B is a non-neuropathic form that is diagnosed in late infancy, childhood or even adulthood, with the majority of type B patients living until late adulthood (McGovern et al., 2008). There are also intermediate forms of the disease that feature delayed neurological onset and a slowly progressive disease course characterized by cerebellar ataxia, extrapyramidal involvement and psychiatric disturbances (Pavlu-Pereira et al., 2005). The clinical presentation of NPD type C is extremely heterogeneous and features both visceral and CNS involvement. Disease onset varies from neonatal to adulthood, and some patients only live a few days, while others may live into their sixth decade. Most patients develop a progressive neurological disease that often features cerebellar ataxia, dysarthria, dysphagia, cataplexy, seizures, dystonia, vertical supranuclear gaze palsy and progressive dementia (Vanier, 2013).

Sphingomyelin, the substrate that accumulates to varying degrees in all NPD patients, is a major structural component of plasma membranes, endosomes and lysosomes. Sphingomyelin is also bioactive in numerous cellular processes including membrane trafficking, cellular migration and proliferation, DNA repair and autophagic death (Taniguchi and Okazaki, 2014). Emerging evidence suggests that altered ASM activity and sphingomyelin cacostasis may also contribute to the pathogenesis of PD, MSA and ALS. For example, some type B NPD patients feature a clinical presentation that resembles PD (Volders et al., 2002). Moreover, sphingomyelin levels are significantly elevated in the substantia nigra of patients with PD (Riekkinen et al., 1975), and mutations in SMPD1/ASM are associated with an increased risk for developing PD (Foo et al., 2013; Gan-Or et al., 2013; Dagan et al., 2015). In the brains of MSA patients, sphingomyelin levels are severely depleted in the white matter (Don et al., 2014), and the expression of the ATP-binding cassette transporter A8, which promotes the synthesis of sphingomyelin, is significantly up regulated (Bleasel et al., 2013). Aberrant changes in sphingomyelin also appear to contribute to the pathogenesis of ALS; disease-related changes in sphingomyelin are present in the spinal cords of sALS patients and SOD1^G93A^ mice (Cutler et al., 2002; Dodge et al., 2015; Henriques et al., 2015).

Mutations in NPC1 and NPC2 genes that impair cholesterol esterification result in the mislocalization of free cholesterol in the CNS of NPD type C patients. Unesterified cholesterol accumulation in NPD type C disease patients, identified by staining with the polyene antibiotic filipin, is a secondary metabolic defect that is also observed in type A disease patients (Sokol et al., 1988), and its clearance from the CNS is routinely used to assess the efficacy of therapeutic intervention in ASM knockout mice (Dodge et al., 2005). Although there are no reports of free cholesterol accumulation in some of the more common diseases of the motor system, cholesterol homeostasis is disrupted in some of these diseases. For example, aberrant cholesterol metabolism is observed in ALS (Cutler et al., 2002), Friedreich’s ataxia (Nestruck et al., 1984), HSP (Tsaousidou et al., 2008), MSA (Lee et al., 2009) and cerebrotendinous xanthomatosis, a disorder that features juvenile cataracts, cognitive impairment, cerebellar ataxia and spastic paraparesis (Sugama et al., 2001).

## Pompe Disease, Alpha-Glucosidase and Glycogen

Glucose derived from glycogen enters the glycolytic pathway to generate pyruvate and subsequently acetyl-CoA, the fundamental building block of cholesterol and fatty acids. Fatty acids are essential for the de novo synthesis of ceramide, cerebrosides (e.g., GlcCer and GalCer), sphingolipids (e.g., sphingomyelin) and glycosphingolipids (e.g., GM3, GM2 and GM1). Therefore, disruptions in glycogen metabolism may adversely affect lipid synthesis or, alternatively, may manifest when lipid synthesis is down regulated during states of lipid cacostasis. Pompe disease is an autosomal recessive NMD caused by a deficiency in acid alpha-glucosidase (GAA), the lysosomal enzyme that catalyzes the hydrolysis of glycogen (Figure 2). GAA is expressed in skeletal muscle, the diaphragm, the heart, the liver, kidneys, and the CNS, in which the highest expression is in neurons of the midbrain, brainstem and the anterior horn of the spinal cord (Ponce et al., 1999). In patients with infantile onset of Pompe disease, the deposition of glycogen in skeletal, visceral and cardiac tissues leads to cardio-respiratory failure and death, usually before the child’s 1st birthday (van den Hout et al., 2003). Delayed myelination and mild cerebroventricular dilation are also observed in infantile-onset Pompe patients (Chien et al., 2006). In a mouse model of Pompe disease, glycogen storage occurs rapidly in the CNS (Sidman et al., 2008), and its accumulation in phrenic motor neurons contributes to respiratory insufficiency (DeRuisseau et al., 2009). The rate of disease progression is much slower in Pompe patients with late-onset disease. However, the disease typically progresses to include limb-girdle muscular dystrophy with diaphragm involvement, eventually leading to wheelchair dependance, respiratory failure and death in adulthood (Hagemans et al., 2005).

Adult polyglucosan body disease (APBD) and Lafora disease (LD) are neurodegenerative disorders that are also directly caused by the aberrant deposition of glycogen. The intracellular accumulation of polyglucosan bodies in the PNS and CNS is the pathological hallmark of APBD, a rare autosomal recessive leukodystrophy caused by a deficiency in glycogen-branching enzyme. Clinically, APBD is characterized by neurogenic bladder, progressive pyramidal paraparesis and polyneuropathy. APBD usually manifests in the 5th or 6th decade of life, slowly progressing until the loss of independent walking, often followed by cognitive decline and premature death (Robitaille et al., 1980; Maegawa et al., 2006). APBD patients are frequently misdiagnosed with multiple sclerosis, ALS or peripheral neuropathies (Hellmann et al., 2015). LD is a fatal neurodegenerative disease that is caused by autosomal recessive mutations in either laforin phosphatase (EPM2a) or malin ubiquitin E3 ligase (EPM2b), which are proteins that regulate the activity of glycogen synthase. Symptoms of LD include seizures, early learning difficulties, dysarthria, ataxia, visual hallucinations and dementia, all of which worsen over time and typically become intractable by 5 years of age (Turnbull et al., 2012; Duran et al., 2014).

Additional diseases with neuromuscular involvement that are also associated with glycogen cacostasis include NPC1, HSP, ALS and Zellweger spectrum disorder (ZSD). For instance, reductions in GAA expression and glycogen storage manifest as secondary metabolic abnormalities in NPC1 (Rauniyar et al., 2015), and phospholipase PAPLA1, associated with HSP, regulates the expression of several regulators of carbohydrate metabolism and glycogen storage (Galiková et al., 2017). Glycogen levels are elevated in the spinal cords of sALS patients, and interestingly, although acidic GAA activity is not altered, neutral GAA activity is up regulated, thus suggesting that glycogen storage occurs outside of the lysosome during the course of the disease. In SOD1^G93A^ mice, glycogen accumulation is the spinal cord is associated with reduced acidic GAA activity; however, elevated neutral GAA activity is also observed (Dodge et al., 2013). Interestingly, in a retrovirus-induced mouse model of motor neuron degeneration, glycogen deposition also occurs in the spinal cord (Brooks et al., 1983). ZSD is a heterogeneous group of genetic disorders that are caused by mutations in peroxin genes that regulate the assembly and function of peroxisomes. The clinical spectrum of ZSD is complex, ranging from profound neurological symptoms in infants to progressive neurodegeneration in adults. CNS-related symptoms of ZSD include seizures, retinal degeneration, hearing loss, psychomotor retardation, leukodystrophy, cerebellar ataxia and peripheral neuropathy (Braverman et al., 2016). Notably, very long-chain fatty acid accumulation, a metabolic hallmark of ZSD, is in some cases also associated with glycogen deposition in the CNS (Goldfischer et al., 1973; Agamanolis and Patre, 1979). Collectively, these diseases suggest that the mislocalization of glycogen in the CNS potentially contributes to lipid cacostasis and a number of NMD phenotypes.

## Conclusions and Future Directions

Determining the pathological contribution of various lipid species in neurodegenerative disorders of the motor system is very challenging. Insights from neuropathic LSDs provide a conceptual framework to guide this effort (Figure 1). For a number of LSDs, the residual function of the mutated protein often affects the onset, nature and severity of the disease phenotype. Thus, new genetic risk factors may be identified for adult-onset diseases of the motor system by screening for heterozygous mutations in proteins that regulate lipid homeostasis in the EALS. The initial intracellular locus of lipid cacostasis is also an important determinant of disease phenotype. For example, a deficiency in lysosomal GBA activity is associated with GD, whereas a reduction in non-lysosomal GBA activity is linked to SCA and HSP. Thus, methods (e.g., high-resolution mass-spectrometry imaging) that permit the localization of the origin of lipid storage to specific organelles are needed. Understanding the locus of lipid storage is crucial as it likely affects the rate that lipid cacostasis spreads between different organelles and various cell types. LSDs also demonstrate that a deficiency in a single lysosomal hydrolase often results in numerous secondary metabolic abnormalities that contribute to disease pathogenesis. Thus, understanding how different lipid classes influence each other in the context of the disease being studied may be necessary to identify the key driver of disease progression. Moreover, in some instances, lipid fluctuations may be compensatory rather than pathogenic in nature. LSDs also illustrate that the physical properties of the affected lipid often determine which cell types are most vulnerable to disease and, therefore, which regions of the CNS undergo degeneration and which phenotypes manifest. Thus, determining the underlying basis and pathological consequences of lipid cacostasis in NMDs will likely provide innovative targets for therapeutic research.

## Author Contributions

JCD wrote the manuscript.

## Conflict of Interest Statement

JCD is an employee of Sanofi.

## Abbreviations

β-GAL: β-galactosidase
ALS: amyotrophic lateral sclerosis
APBD: Adult polyglucosan body disease
ASAH1: acid ceramidase
ASM: acid sphingomyelinase
AT: ataxia telangiectasia
B4GALNT1: beta-1,4-N-acetyl-galactosaminyl transferase 1, GM2 synthase
CMT: Charcot–Marie–Tooth disease
CNS: central nervous system
EALS: endosomal-autophagic-lysosomal system
ER: endoplasmic reticulum
FA2H: fatty acid 2- hydroxylase
FD: Farber disease
FVT1: 3-ketodihydrosphingosine reductase
GAA: Acid alpha-glucosidase
GALC: galactocerebrosidase
GalCer: Galactosylceramide
GBA: glucocerebrosidase
GBA2: non-lysosomal GBA
GBS: Guillain-Barré syndrome
GD: Gaucher disease
GlcCer: glucosylceramide
GLD: globoid cell leukodystrophy
HIBM: hereditary inclusion body myopathy
HMSN: motor and sensory neuropathy
HSCT: hematopoietic stem cell transplantation
HSN1: hereditary sensory neuropathy type 1
HSP: hereditary spastic paraplegia
IBMPFD: Paget’s disease of bone, frontotemporal dementia
KD: Krabbe disease
LD: Lafora disease
LSDs: Lysosomal storage diseases
MMN: multifocal motor neuropathy
MND: motor neuron disease
MSA: multiple system atrophy
NBIA: neurodegeneration with brain iron accumulation
NCLs: neuronal ceroid lipofuscinoses
NPD: Niemann-Pick disease
PD: Parkinson disease
PMD: Pelizaeus–Merzbacher disease
PNS: peripheral nervous system
SMA-PME: sphingomyelin phosphodiesterase 1(SMPD1), spinal muscular atrophy myoclonic epilepsy
SPTLC1: serine palmitoyltransferase long chain base subunit-1
VCP: valosin-containing protein
ZSD: Zellweger spectrum disorder.
